# Patients’ experiences of seeking help for emotional concerns in primary care: doctor as drug, detective and collaborator

**DOI:** 10.1186/s12875-020-01106-z

**Published:** 2020-02-14

**Authors:** Daisy Parker, Richard Byng, Chris Dickens, Rose McCabe

**Affiliations:** 1 College of Medicine and Health, University of Exeter, Exeter, UK; 2 Plymouth University, Plymouth, UK; 3 City, University of London, London, UK

**Keywords:** Mental health, Primary care, Professional-patient relationship, Qualitative research

## Abstract

**Background:**

NICE guidelines for the management of emotional concerns in primary care emphasise the importance of communication and a trusting relationship, which is difficult to operationalise in practice. Current pressures in the NHS mean that it is important to understand care from a patient perspective. This study aimed to explore patients’ experiences of primary care consultations for emotional concerns and what patients valued when seeking care from their GP.

**Methods:**

Eighteen adults with experience of consulting a GP for emotional concerns participated in 4 focus groups. Data were analysed thematically.

**Results:**

(1) Doctor as Drug: Patients’ relationship with their GP was considered therapeutic with continuity particularly valued. (2) Doctor as Detective and Validator: Patients were often puzzled by their symptoms, not recognising their emotional concerns. GPs needed to play the role of detective by exploring not just symptoms, but the person and their life circumstances. GPs were crucial in helping patients understand and validate their emotional concerns. (3) Doctor as Collaborator: Patients prefer a collaborative partnership, but often need to relinquish involvement because they are too unwell, or take a more active role because they feel GPs are ill-equipped or under too much pressure to help. Patients valued: GPs booking their follow up appointments; acknowledgement of stressful life circumstances; not relying solely on medication.

**Conclusions:**

Seeking help for emotional concerns is challenging due to stigma and unfamiliar symptoms. GPs can support disclosure and understanding of emotional concerns by fully exploring and validating patients’ concerns, taking into account patients’ life contexts. This process of exploration and validation forms the foundation of a curative, trusting GP-patient relationship. A trusting relationship, with an emphasis on empathy and understanding, can make patients more able to share involvement in their care with GPs. This process is cyclical, as patients feel that their GP is caring, interested, and treating them as a person, further strengthening their relationship. NICE guidance should acknowledge the importance of empathy and validation when building an effective GP-patient partnership, and the role this has in supporting patients’ involvement in their care.

## Background

General Practitioners (GPs) are the most frequently used providers of mental health care in the UK [1], with up to 40 % of consultations having an emotional or psychological agenda [2]. The mental health problems faced in primary care are heterogeneous, undifferentiated, and present as a continuum with symptoms of different diagnoses often inextricably linked [3, 4]. Due to this complexity, this study uses the term ‘emotional concerns’ throughout to reflect the patients most commonly seen by GPs.

Care for emotional concerns is usually informed by NICE guidelines, which state that doctors should “*build a trusting relationship and work in an open, engaging and non-judgemental manner*” [5, 6]. There is a consensus that a good relationship and communication are central to person-centred care. Previous literature examining patients’ experiences of seeking care for emotional concerns have found that patients prioritise GPs’ interpersonal skills over the effectiveness of treatment [7, 8]. Patients value being listened to, given time to talk [9, 10] and expect their GP to help them open up and explore their experiences [9–11]. The doctor patient relationship is central, and is underpinned by empathy, feeling understood, and being known as a person [9, 12, 13].

However, a therapeutic relationship can be difficult to operationalise in practice and challenging in the current climate of huge demand on primary care services. Consultations lasting on average eight minutes and difficulties seeing the same GP mean that lack of time and continuity [14, 15] are substantive barriers to developing a therapeutic relationship. Stigma associated with emotional concerns creates barriers to seeking help and disclosing concerns, with patients often presenting their emotional agenda late in the consultation, leaving little time for the doctor to explore these concerns [16–18]. The symptoms associated with emotional concerns – demotivation, indecision and uncertainty, guilt - can further problematise patients’ ability to articulate their experiences, for example by not fully describing concerns, minimising concerns and feeling guilty about taking up the doctor’s time [10].

These problems point to the lack of time in GP consultations and the consequences of attempting to address emotional concerns without the time and resources to develop a trusting relationship, a shared understanding of the concern, and engage patients in treatment plans. As a result, it is important to understand how patients experience care within the current climate of limited resources in the NHS, in order to understand aspects of care that work well, and aspects that work less well.

Therefore, the aim of this study was to explore patients’ experiences of seeking help for emotional concerns in primary care, with view to understand best practice from a patient perspective within the constraints of busy clinical practice.

## Methods

### Terminology

In GP consultations, mental health concerns may be understood by GPs and patients in various ways and also encompass a broader range of problems than diagnosed mental health disorders. Hence, in this study, the term ‘emotional concerns’ is used to represent this diversity of experiences and understandings across patients and practitioners and includes; 1) common mental health problems, specifically anxiety and depression, 2) undifferentiated low mood, stress and/or anxiety that may be sub-clinical or not formally diagnosed, 3) low mood, stress, and anxiety that may be attributed to difficult life circumstances.

### Design

The study is part of a wider project that aims to develop an intervention to support GPs when communicating with patients with emotional concerns. Focus groups were used to facilitate the unearthing of topics that were not previously considered by the researchers. Compared to individual interviews, focus groups have a more naturalistic interaction and group dynamics can facilitate disclosure when exploring sensitive topics [19–21]. Participants have been shown to feel empowered and supported in a group situation and participants can provide reassurance to one another that would not be possible in an individual interview [20, 21]. Focus groups allow participants to build on each other’s contributions or challenge each other’s statements, leading to the production of more elaborate accounts than would be gained by doing individual interviews [22].

### Recruitment

An email introducing and describing the study was sent to one service user group which consists of 18 individuals with lived experience of emotional concerns who are commonly involved in research. Additionally, posters were also displayed in and around University of Exeter campuses, a local sports centre, and in a local mental health support centre. All recruitment was conducted in Devon. Recruitment sites were purposively targeted to allow for variation in patients’ socioeconomic status and education, and to target hard to reach participants. Posters briefly described the study and outlined that individuals with lived experience of “seeking help from their GP for emotional or mental health concerns” were being recruited. Participants self-selected by emailing DP or by attending a pre-arranged focus group.

Participants were included if they reported experience of seeking help from their GP for emotional concerns, were able to give informed consent, and considered themselves to be psychologically well enough to participate. Participants were not recruited based on diagnostic criteria in order to ensure that potential participants who did not identify with the diagnostic labels of depression and anxiety were not excluded. The time between the patient seeking help from their GP and them participating in the focus group was not specified.

The email explained that the study would involve attending one focus group to explore patients’ experiences of seeking help for emotional concerns from their GP. Ethical approval was granted by the University of Exeter Medical School Research Ethics Committee (Reference: 16/11/111) prior to the commencement of the study.

### Procedure

Focus groups were conducted between March and August 2017. Participants in groups one and four were familiar to each other, whereas participants in groups two and three were strangers. Participants were given a detailed information sheet about the study before giving consent. The information sheet included information on the purpose of the study, what taking part involved, risks and benefits of participation, how their data would be kept confidential and the participants’ right to withdraw. Written informed consent was taken by DP before the start of the focus group. Three focus groups were conducted at the University of Exeter and at one local mental health support centre. Participants took part in one focus group with between three to five other participants. Four focus groups were conducted in total. The groups were facilitated by DP and a second researcher acted as co-facilitator. All focus groups were audio-recorded using two digital voice recorders. Participants were informed of their right to withdraw from the study at any time. Due to the potentially distressing nature of the topic, a standardised risk assessment protocol was in place should participants become distressed. The risk assessment protocol was to be used if any participant disclosed thoughts of self-harm and included standardised questions and a flowchart of actions questions to assess and manage risk of self-harm. Fortunately, no participants became distressed during, or as a result of, the focus groups.

### Topic guide

The discussion followed a semi-structured topic guide which was designed to elicit areas of interest whilst also allowing participants to expand on their narratives and topicalise areas of personal importance. Questions were designed to allow participants to give a free narrative and build on one another’s responses. The topic guide was developed for this study and was based on the aims of the research and focused on two key areas: 1) patients’ experiences of seeking help from their GP for emotional concerns, including whether they were satisfied with the care given, anything they would have changed, and what was done well, and 2) what aspects of care patients particularly valued, including their opinions on what makes an ‘ideal’ consultation and their perceived barriers to this. The topic guide was iteratively developed based on evidence from previous studies about mental health in primary care, and clinical and research experience of the research team. A copy of the topic guide is presented in additional file 1.

### Data analysis

Data collection and analysis was conducted concurrently, so that early insights could inform the focus of later focus groups. This also enabled us to gauge the richness of the accounts, which informed decisions about sample size. As these focus groups were being conducted with a potentially vulnerable population and had the potential to cause distress, we did not want to conduct more groups that was necessary. Therefore, when few new insights were being generated, we ceased recruitment.

Focus groups were transcribed verbatim and anonymised. Transcripts were analysed using inductive, reflexive thematic analysis in accordance with guidelines recommended by Braun and Clarke [23, 24]. Transcripts were organised and managed using qualitative data analysis software NVivo 11 [25]. All transcripts were initially analysed independently by DP, a PhD student with 3 years of qualitative experience in healthcare and psychology. First, familiarisation with the data was achieved by transcribing and checking the transcripts. Secondly, all of the transcripts were coded line-by-line. The codes identified features of the data (semantic, content, or latent) that may form the basis of repeated patterns. In line with the inductive approach taken, we gave full and equal attention to each data item to enable us to develop a rich and nuanced analysis [23].

Next, these codes were organised into categories which were considered in the context of the wider transcripts. Categories were developed by grouping codes by what topics and processes clustered together and which were distinctly different. This allowed for the initial organisation of the codes into patterns of shared meaning across the data. These categories were iteratively refined using a constant comparative process, moving from descriptive categories to conceptual themes and subthemes. Maps and diagrams were used throughout to interrogate the relationships between themes. Codes and categories were printed out and discussed in series of meetings with RM, RB, and two individuals with lived experience throughout to develop consensus about the analysis and ensure reliability of the analysis. Data was also presented at regular qualitative data sessions. Discussing the analysis with wider groups allowed for the development of a more nuanced, rich, and in-depth analysis. This approach also guarded against individual idiosyncratic of highly subjective interpretations of the analysis [26].

## Results

Thirty-two individuals responded to advertisements. Fourteen individuals were not available on the days of the focus groups. Four focus groups were conducted lasting an average of 96 min each. Eighteen patients participated. All attending participants were psychologically well enough to participate and to provide written informed consent. Participants ranged in age from below 25 to over 50. Participants from disadvantaged areas were well represented. Participants’ reported diagnoses included depression, anxiety, OCD, and learning disabilities, however these were not formally collected to avoid excluding participants who did not identify with, or who did not want to disclose, a psychiatric diagnosis. Participants’ sociodemographic characteristics and detail of the focus groups are presented in Table 1 and Table 2.

**Table 1:** Sociodemographic Characteristics of Participants

Sex	
Women	10
Men	8
Recruitment Site	
Service user involvement group	4
Community	10
Mental Health Support Centre	4

**Table 2:** Participant Characteristics and Length of Focus Groups

	Participants	Female Participants	Male Participants	Length (minutes)
Focus Group 1	4	2	2	102.40
Focus Group 2	6	3	3	108.24
Focus Group 3	4	2	2	101.26
Focus Group 4	4	3	1	73.37
Total	18	10	8	385.27

### Findings

**Fig. 1:**
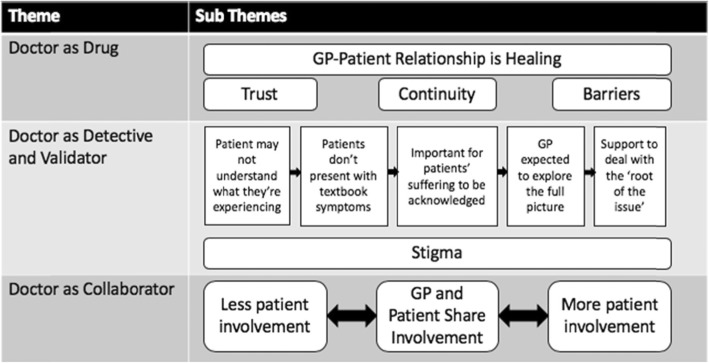
Three Overarching Themes and 13 Sub Themes

Line by line coding of transcripts generated 343 codes. Codes were then grouped into 29 categories, which were refined and revised to generate 13 sub-themes and three overarching themes: doctor as drug; doctor as a detective and validator of emotional concerns; and doctor as collaborator. Themes and sub-themes are represented in Fig. 1.

#### Doctor as drug

For patients experiencing emotional concerns, the relationship with their GP was intrinsically healing. Some patients resisted medical support, such as medication, in favour of a connection with their doctor.

“What makes an excellent clinician in my experience, it’s not just being able to treat things amazingly and do great surgery, it’s about the relationship with the people, because that’s what helps us get better is that relationship.” (P11, male)

While a good doctor-patient relationship was important for all medical care, a human approach was particularly important for patients experiencing emotional concerns. In particular, patients wanted GPs to be empathetic, listen attentively, and understand their problems.

“If you had flu, they’d give you medication for that. [GPs] don’t emphasise with you and go ‘wow must be really hard having flu’ they just say ‘oh you’re unwell here’s some medication’. I feel like with mental health, in my experience, it can be a similar approach. But, because of the nature of mental health, it’s a difficult subject to talk about, and because of the effect that it can have on you emotionally and all aspects of your life I think you need to have a conversation which allows you to actually say that and be heard and understood, otherwise it might make you reluctant to go and call for help.” (P13, male)

Patients valued a long-term relationship with their GP, however, it was hard to maintain continuity due to difficulties accessing appointments. Patients were grateful for GPs who prioritised personal continuity by booking follow up appointments for them. Continuity with a GP was particularly important when patients were on a waiting list for psychological therapy, which were often very long, or when they were starting medication.

“I had a very positive experience at the doctors, he said I’m going to put you on a cognitive behaviour therapy course, unfortunately there is a six week wait but I’ll make an appointment for you in a fortnight to come back to me.” (P8, female)

However, one patient described how they would rather see a GP they didn’t know, as the anonymity helped them to open up about their difficulties, about which they were ashamed.

“[It’s] not to do with my relationship with the doctor that I usually saw I thought she was a great doctor she’s been very empathetic and someone that I felt like I could talk to, I just felt, because I think I was ashamed about what I wanted to say so having someone that … ” (P12, female)

“Didn’t know you at all.” (P13, male)

“Yeah I think just helped slightly.” (P12, female)

Many patients were ashamed about what they wanted to say, and as a result, trusting their GP was paramount. Patients were more likely to follow their GP’s advice if they trusted them.

“When he did suggest that I go on the tablets, because I’d built up confidence in him, I thought no I’m going to give it a go.” (P8, female)

Time pressure presented a barrier to the doctor-patient relationship. Many patients felt that their care was rushed and impersonal, but patients conceded that GPs were doing the best they could with limited resources.

“I’ve had the situation where I was telling the GP something and literally she was stood with the door wide open you know off you go ten minutes are up.” (P17, female)

Finally, the GP’s computer and desk can be a barrier to the therapeutic relationship. Note taking was a distraction, both for the GP and for the patient, who worried about what the GP was recording.

“My own experience is when I go to the GP, and the thing that’s actually the big thing is this dirty great computer just here and you’re going to be doing this [typing] whilst talking to me and that means I’ve only got about sixty percent of your attention … it is also a huge distraction when you just want to have a therapeutic supportive conversation.” (P11, male)

#### Doctor as detective and validator of emotional concerns

For consultations with an emotional focus, GPs were expected to take on the role of detective and validator. This was different to a physical health problem which could be considered in a more isolated way from the patient and their context. The experience of emotional concerns was alien to some patients, and patients often presented to their GP with puzzling, non-textbook, or somatic symptoms. Patients expect their GP to be effective at picking up cues and eliciting their emotional concerns.

“I think that [the first] conversation needs to be something just to help people understand what exactly it is that’s happening to them, because it’s alien … You think you’ve completely lost the plot.” (P2, female)

Seeking help is further confounded by the experience of stigma. Patients found it hard to seek help, and when they do seek help, they find it difficult to open up to their GP and often need a ‘run up’, which may be starting the consultation with a physical concern. It is helpful for GPs to reassure patients and normalise their symptoms.

*“That was huge to me to know that those things were actually normal of depression and I wasn’t going mad and there was a reason why I feel like that.” (P1, female).*

When a patient discloses concerns to their GP, it was important for their GP to acknowledge and understand their suffering. For some patients this may be in the form of a diagnosis that is explained to them.

“I found a diagnosis quite comforting in a weird way because for me it felt like this is a thing I can- as long as you know what you’re dealing with you know that there is a way to treat it and to cope with it” (P13, male)

When patient’s concerns were not acknowledged, they felt that their concern was not important, or that there was no point to seeking help.

*“You need someone to understand you and say yeah, I recognise what you’re going through it sounds like hell.” (P13, male).*

Patients wanted to explore the possible causes of their emotional concerns, which were often believed to be stressful life events. However, other patients did not think that their GP acknowledged the role of life circumstances.

“I think maybe they missed out on not diagnosing why I felt the way I’m feeling, they could have said “look the way you’re feeling is actually quite understandable because you lost this really important thing to you it’s quite understandable that you’re not feeling okay”.” (P13, male)

When patient’s full story was not explored, they felt that their emotional concerns were managed on a superficial level that did not deal with the root of the problem. For these patients, being prescribed medication was considered a ‘quick fix’. Many patients did not want to take antidepressants, often due to fears about addiction, but discussing this with their GP could attenuate their fears. Patients were also more willing to take anti-depressants if their GP had considered how best to help them as an individual. Some patients preferred to develop coping mechanisms, which were empowering and a long-term solution, over taking an antidepressant, which was seen by some as an artificial cure.

*“I felt it was important to go through the experience of what I was feeling in order to complete the process of healing, I felt that if I was taking antidepressants, whilst it would lift my mood and make me feel able to cope, I would also suppress what was going on and if I suppress that it means I wouldn’t be able to deal with it.” (P11, male).*

#### Doctor as collaborator

The amount involvement patients wanted to have in their care. Different patients preferred different levels of involvement, and patients preferred different levels of involvement at different stages of their care experience. Specifically, patients varied in the levels of involvement they preferred take when making decisions about treatment. Patients also varied in their ability to be proactively engaged in certain treatments and referral pathways.

GPs were expected to accurately judge how much involvement a patient was able to have, and then meet them at that point.

“An experienced practitioner would be able to tell what the patients’ needs are quite quickly from gauging them, and then go ‘this person will actually get back on their feet, I need to make some advice and I know that they will take it and I trust that in two weeks’ time when I speak to them again they will have actually followed up on this advice whereas this other patient I feel actually I do need to maybe try and check in with them a bit sooner or make a step for them because I don’t think they’re quite capable of making that step themselves now but they do need this help quite quickly’” (P11, male)

For most patients, the ideal scenario was sharing involvement with a GP, where both doctor and patient expertise were utilised. One way GPs supported patients to be move involved was providing self-care tips.

*“[my GP] used to give me some print offs from the computer, wellbeing tips, like go for a walk every day.” (P6, male).*

However, for others this advice was perceived as patronising and ill-fitting to their level of distress. Self-care advice may be interpreted as the GP has not understood their emotional concerns and is not doing enough to support them.

“The GP did actually [recommend online CBT]. Oh god am I really going to go through that when I can’t even get out of bed and I can’t sleep. Some of the advice that the GPs give you is actually really condescending.” (P1, female)

Collaboration and an equal partnership were particularly important when making decisions about antidepressants. Being involved in these discussions made patients feel that their treatment is tailored to them and their needs.

*“[My GP] did have that discussion with me and said to me you know this is what we can start you on, so I felt quite happy.” (P2, female).*

A collaborative partnership could be encouraged by providing patients with accessible information about their emotional concerns, medication, sources of support and length of waiting lists. One way to convey this information is using leaflets. Most patients accepted leaflets, especially if the resources were clear, plainly written, and printed out. However, the way that leaflets are presented to patients is important. Patients would reject leaflets that were seen as a substitute for active support.

“It’s how they present that to you … I’ve had lots of leaflets. [GPs] have to say to them “this is a bit of information for you when you’re feeling like it have a read through”.” (P1, female)

While an equal partnership between the doctor and patient was preferred, often patients felt that they needed to take control over their care in the form of ‘managing’ their GP. Some patients researched their diagnosis so that they could take an active role in discussions with their GP, whereas other patients used their GP as a gatekeeper. These patients go to the GP not for emotional support, but for practical resources such as a referral, medication, or a sick note.

*“I have to become the expert because my experience has been that you tend not to get very good information from the GP.” (P10, male).*

However, other patients wanted their GP to take more control over their care. For some of these patients, the symptoms of their emotional concerns meant that they felt unable to take an active role in their care. This was particularly pertinent when patients had to self-refer to psychological therapy. Making phone calls was challenging for many patients; demotivation and hopelessness meant that it was not ‘just’ a phone call but an insurmountable challenge.

“Sometimes your head is in such a mess and you feel so overwhelmed and can’t cope that you actually need the doctor to go “I’m going to call them and ask them to call you”.” (P13, male)

##### How themes interrelate

**Fig. 2:**
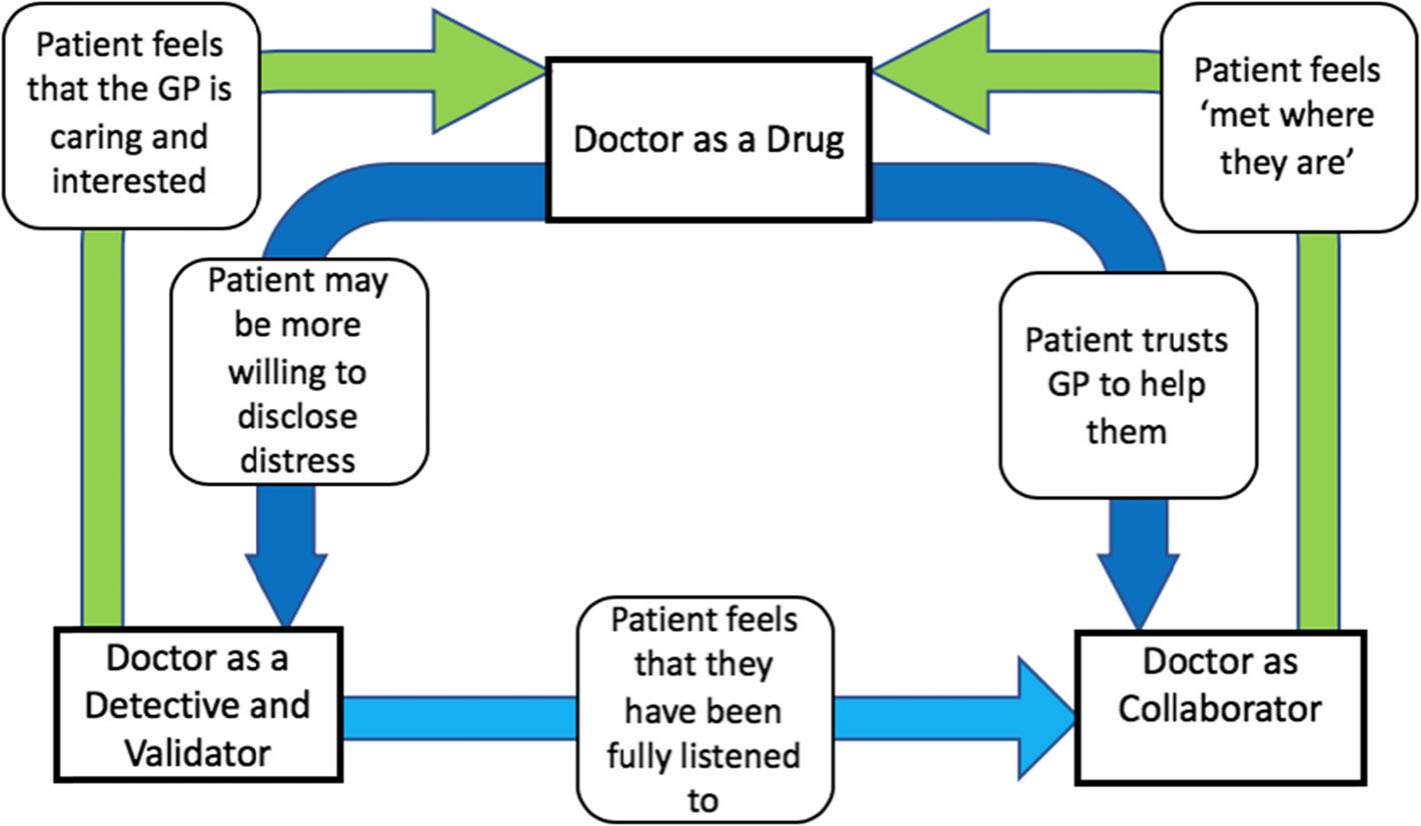
Mechanisms Underpinning Effective Consultations for Psychological Distress in Primary Care

The three themes interrelate in key ways, as outlined in Fig. 2. A therapeutic relationship is the foundation of mental health care as it facilitates trust and helps patients open up. The consultation feeds back into improving this relationship. Feeling listened to and that one’s experiences have been fully explored makes the patient feel that their GP is caring and interested. When the GP is sensitive to patient’s ability and willingness to be involved in their care, and adapts their care appropriately, the patient feels met.

The model in Fig. 2 shows how shifts in the ethic and mode of practice are important and possible, and this study has identified specific tactics to underpin these new modes of working, outlined in Table 3.

**Table 3:** Patient reports of ‘what works well’

Theme	What Works Well	Quotes
Doctor as Drug	GPs can support patients by booking their follow up appointments for them.	SU1: *“Come back I’ve made you an appointment we’ll see you at ten o’clock next week”, that’s what you need [GPs to do].*
	Where it is not possible to have a long-term relationship, GPs can quickly build rapport in a number of ways, such as calling the patient by their name and not attending to a computer.	*SU3: he’s still charming and lovely and shakes hands and all the rest of it and calls me by my first name and smiles.* *SU11: [the computer] is a huge distraction when you just want to have a therapeutic supportive conversation .*
Doctor as Detective and Validator	Give an explicit verbal acknowledgement of the patients’ emotional problems.	*SU13: you need someone to understand you and say “I recognise what you’re going through it sounds like hell.”*
	Work with the patient to help them understand what is happening to them.	*SU2: I think the first [consultation] needs to be something just to help people understand what exactly it is that’s happening to them because it’s alien.*
Doctor as Collaborator	Make self-referral phone calls to Improving Access to Psychological Therapy for patients who feel unable.	*SU11: I would have appreciated it if someone had actually picked up the phone and called [local depression and anxiety service] for me.*
	Provide self-care resources for patient who feel able to engage with them.	*SU4: [I was given] a checklist of things to look at of things which I knew would help me if I started to go down and that included classic things like exercise getting out with mother nature.*
	Share information about treatment options, side effects to help patients engage, but present resources sensitively.	*SU10: basic information on the mental health services, what is the treatment for your particular condition, how long is the waiting list, what do you do on that period on the waiting list when don’t have any support. Those would be useful things* *SU1: it’s how they present that to you … [GPs] have to say “this is a bit of information for you when you’re feeling like it have a read through”.*

## Discussion

### Summary

Three themes were generated. Firstly, patients considered their relationship with their GP to be intrinsically therapeutic. Continuity of care was particularly valued, but there were often barriers to this. Secondly, patients expected their GP to act as a detective and validator. Puzzling symptoms often meant that patients did not understand their emotional concerns. This and stigma made disclosure challenging. GPs needed to play the role of detective by exploring not just symptoms, but the person and their life circumstances. GPs were crucial in helping patients understand and validate their emotional concerns. Finally, patients prefer a collaborative partnership with their GP, but often need to relinquish involvement because they are too unwell, or take a more active role because they feel GPs are ill-equipped or under too much pressure to help.

### Strengths and limitations

Participants were of varying ages and from different backgrounds and males and females were both well represented, allowing for a range of experiences to be heard. The main limitation of this study was that the sample may not be generalisable. The sample may be biased in a number of ways.

Firstly, participants were self-selecting and therefore may have been more likely to be pro-active and engaged in their care. It may be the case that participants presented experiences from the extreme ends of the spectrum, as participants with less note-worthy experiences may be less likely to participate. It is also a risk that dominant individuals in groups may lead the discussion. However, question guides were designed so that all participants could contribute, and the facilitator encouraged less vocal members of the groups to share their views.

Secondly, there are a number of barriers to seeking help from a GP for emotional concerns, including poverty and poor psychological literacy [27]. As only participants who had sought help from their GP were recruited, individuals who experienced emotional concerns and were not able to seek help from a GP will have been excluded. In addition, the sampling strategy used may advantage participants who are well equipped to discuss their experience. Individuals who chose to participate in this study may have a level of insight that other participants may not have had. However, participants were recruited from various locations in the South-West of England in order to maximise the recruitment of hard to reach participants.

As participants were recruited in the South-West of England, it was difficult to recruit an ethnically diverse sample. A broader recruitment area and using maximum variation sampling may have reduced this limitation and should be considered for future research.

Finally, there is a risk of recall bias when exploring patients’ experiences of past events. As we did not control for the amount of time between the patient seeking help from their GP, and them participating in the focus groups, there is a risk that participants may not accurately remember their experiences of seeking help from their GP. Targeting participants who have sought help from their GP recently may have reduced this limitation.

### Comparison with existing literature and implications for practice

These findings have practical implications for GPs. Patients in this study discussed the importance of developing and maintaining a relationship with their GP. This finding is reflected in the NICE guidelines, which highlight the importance of a trusting relationship when supporting patients with emotional concerns such as depression and anxiety [5, 6]. Previous studies reiterate the importance of the therapeutic relationship to patients [12, 14], which is associated with improved shared understanding [28], treatment adherence [29, 30] and improved treatment outcomes [31, 32].

However, guidelines about the GP-patient relationship are often poorly defined and it is not clear how this relationship can be developed and maintained in practice. Patients in this study highlight suggest that demonstrating empathy, validation, and concern can contribute to the development of this relationship [12, 14, 33–35]. Another important component of this relationship was being attentive. Where possible, GPs should avoid attending to their computer, as this is interpreted by patients as a disengagement of attention and a sign of disinterest [12, 35–37]. Finally, maintaining the continuity of this relationship was also important. Patients suggested that GPs could maintain their relationship with patients by booking follow up appointments for them. This conveys a personal interest in the patient and allows for more time which prevents GPs from appearing rushed.

Patients in this study also discussed how GPs could help them to understand, validate and normalise their emotional concerns. To explore patients concerns and help them to open up, GPs could display interest and understanding [14] and ask direct questions [15, 35]. After their concern has been explored, patients want GPs provide an explanation for their emotional concerns. This may be in the form of a diagnosis, or simply giving information on the cause, course and prognosis of the concern [11]. Eliciting the patients’ understanding of the nature and cause of their emotional concerns is valuable, as this will affect a patients’ treatment preferences and adherence [38].

Finally, shared decision making is increasingly highlighted in the literature and guidance [5, 6]. This study has contributed to understandings about the effectiveness of shared decision making in practice. An equal and collaborative relationship was considered ideal by patients in this study. A preference for health care practitioners to act as a ‘guide, not a director’ is highlighted in previous research [12]. Increasing collaboration improves patients’ symptoms and may improve the doctor-patient relationship, as this demonstrates to patients that their opinions are valid [39].

However, patients in this study reflected differences in their willingness and capability to be involved in the their care. Similarly, Benbassat [40] found that patients’ preferred model of involvement is multidimensional. For example, some patients may be more involved in information seeking, but prefer less of a role in treatment decisions. As a result, it is important for clinicians to inquire about patients’ preferences directly [40].

For patients who are less able to be involved their care, GPs can support them by making referrals to psychological services for them. For patients who are able to be more involved, this can be facilitated by providing them with self-care strategies and clear written information. However, this needs to be done alongside a full exploration of the patients concerns, as giving patients information or advice before their concerns have been fully elicited leads to patients not feeling understood [9, 36].

## Conclusion

Previous studies have highlighted the role of the GP-patient relationship when supporting patients with emotional concerns in primary care. This study builds on these findings by outlining why this relationship is important in relation to other processes in the consultation. Seeking help for emotional concerns is challenging due to stigma and unfamiliar symptoms. GPs can support patients to disclose and understand their emotional concerns by fully exploring and validating patients’ concerns, taking into account the patient’s life context. This process of exploration and validation forms the foundation of a curative, trusting GP-patient relationship. A trusting relationship, with an emphasis on empathy and understanding, can make patients more able to share involvement in their care with their GP. This process is cyclical, as patients feel that their GP is caring, interested, and treating them as a person, further strengthening their relationship. These findings have implications for practice by adding to understanding about how a GP-patient relationship can be developed in practice, and by highlighting patients’ varying preferences for involvement in decisions. Therefore, NICE guidance [5, 6] should acknowledge the importance of empathy and validation when building an effective GP-patient partnership, and the role this has in supporting patients’ involvement in their care.

## Additional File

**Additional file 1:** Topic guide for focus groups.

## Data Availability

Due to the sensitive nature of the data, authors do not have consent to make data available.

## Abbreviations

GP: General Practitioner
NHS: National Health Service
NICE: National Institute for Health and Care Excellence
