# Supplementary material for: Patients’ experiences of seeking help for emotional concerns in primary care: doctor as drug, detective and collaborator
Source: BMC Fam Pract. 2020 Feb 14;21:35. doi: 10.1186/s12875-020-01106-z (PMC7020382; doi:10.1186/s12875-020-01106-z)
Supplement: Supplementary file 1 — Additional file 1: Topic guide for focus groups. [file 12875_2020_1106_MOESM1_ESM.docx]

**Focus Group Topic Guide**

**Welcome:**

Good morning and welcome to our session. Thank you for taking part in this focus group, we appreciate your time. My name is Daisy Parker and I am a PhD student at the University of Exeter Medical School.

Today we will be discussing your experiences of seeking help from your GP for emotional concerns and your ideas about how things can be improved. The information from these focus groups will be used to inform the development of an intervention to give GPs some extra skills in communicating with patients with mental health concerns. We will start by talking about your experiences, and then in the second half of the focus group we will focus more on ideas about how treatment can be improved. Please contribute as much as you like to this discussion. There are no wrong answers and we are keen to hear all of your opinions.

As explained in your participant information sheets, these sessions are being audio recorded. However, what is said in this group will remain anonymous. To promote confidentiality, I will ask that you are allowed to discuss what was said in the group with others but nothing should be done to identify, either directly or indirectly, who said what.

During the group, talk amongst yourselves but please try to speak one at a time. There are no right or wrong answers and you do not have to agree with each other. I want to hear from all of you.

If you feel uncomfortable or distressed at any time, please feel free to not answer a question or to leave the group. The co-facilitator will go with you to make sure that you are okay. There will be no adverse consequences to you leaving early.

Does anyone have any questions before we start?

**Warm up:**

What’s your name (you don’t have to give us your real name) and if you could only eat one thing for the rest of your life what would it be?

**Main Discussion: Current Model**

1. Think back to your first experience of going to your GP about emotional concerns. What was it like?
   1. Were you satisfied the consultation? Why/ why not?
   2. Is there anything you would have liked to have been done differently?
2. In general, how do you feel about the way emotional concerns are managed in GP consultations?
   1. What is done well?
   2. Are there any issues?
3. Has a GP has said or done that has been particularly helpful?

**Improving Practice**

1. Did you receive a diagnosis? What were your thoughts on this?
2. Did you receive antidepressant medication? What were your thoughts on this?
3. Did you receive a referral? What were your thoughts on this?
4. Did the doctor explore any social factors contributing to your depression?
5. Did you feel able to say everything you wanted to say?
6. If money were no object, what do you think an ideal consultation would be like?
7. What do you think it is about primary care that might make these consultations difficult?

Doctor factors? Patient factors? Institutional factors?

**Concluding questions**

Is there anything that we haven’t discussed today that you feel is important?
